# Exosomes Derived From Epigallocatechin Gallate-Treated Cardiomyocytes Attenuated Acute Myocardial Infarction by Modulating MicroRNA-30a

**DOI:** 10.3389/fphar.2020.00126

**Published:** 2020-02-26

**Authors:** Chan Zhang, Xiaowen Gan, Ronggan Liang, Jie Jian

**Affiliations:** ^1^ Department of Pharmacology, Xiangya Hospital, Central South University, Changsha, China; ^2^ Department of Pharmacology, Guilin Medical University, Guilin, China

**Keywords:** acute myocardial infarction, epigallocatechin gallate, exosomes, microRNA-30a, apoptosis, autophagy

## Abstract

**Background:**

Ischemia-derived exosomes can restrict excessive autophagy by transferring microRNA-30a (miR30a) to cells. Reports have confirmed that epigallocatechin gallate (EGCG) alleviates acute myocardial infarction (AMI) by regulating autophagy; however, research evaluating the communication with cardiomyocytes and exosomes is lacking. This study aimed to explore whether exosomes derived from EGCG-treated cardiomyocytes mitigated AMI by adjusting miR30a to inactivate apoptosis and autophagy.

**Methods:**

Exosomes were extracted from cardiomyocytes, cultured either in control or AMI condition, with or without EGCG pretreatment. The exosome characteristics were analyzed by nanoparticle tracking analyses and transmission electron microscopy. The change in miR30a in cells and exosomes was demonstrated by qRT-PCR. H9c2 or stable miR30a knockdown (miR30a^KD^) cell lines were incubated with exosomes derived from EGCG-treated cardiomyocytes *in vitro* or *in vivo*. The effect of EGCG and exosomes on I/R-induced cardiomyocyte apoptosis and autophagy was assessed.

**Results:**

EGCG improved the activity of cardiomyocytes, and increased average diameter, concentration, miR30a mRNA level, and specific protein expression in AMI-derived exosomes produced by cardiomyocytes. Moreover, the coincubation of AMI cells with EGCG or exosomes derived from EGCG-treated cardiomyocytes attenuated cardiomyocyte apoptosis and autophagy.

**Conclusions:**

The findings showed that EGCG upregulates miR30a, which was efficiently transferred *via* exosomes between cardiomyocytes, thereby contributing to the suppression of apoptosis and autophagy. By focusing on the cardiomyocyte microenvironment, we identified a new target of EGCG alleviating AMI by regulating apoptosis and autophagy.

## Background

Exosomes are derived from polycystic vesicles formed by intracellular lysosomal microparticles, carrying various signaling molecules and shuttling from the surface membranes of most cell types. A growing body of evidence has indicated that exosomes play a vital role in intracellular communications and material transport (Li et al., 2017). For example, tumor-derived exosomes deliver tolerogenic signals to immune cells, inhibit immune cell proliferation, as well as induce apoptosis and immune suppression through a paracrine effect (Cheng W. et al., 2019). Neuronal exosomes may propagate between the central nervous system and peripheral circulation, providing neuroprotection against neurological disorders (Shi et al., 2019). A recent study revealed that the release of exosomes may protect the myocardium from cell death (Davidson and Yellon, 2018). However, the underlying mechanism of exosomes in heart disease is not yet fully understood. Hence, further research should be performed with a focus on exosome-mediated cardioprotection.

Acute myocardial infarction (AMI) is a sharp decrease or interruption of blood supply due to coronary artery embolization, resulting in severe and prolonged myocardial tissue ischemia and myocardial cell necrosis (Tamis-Holland et al., 2019). Consequently, alleviating the damage to cardiomyocytes can be utilized as a therapeutic strategy in AMI injury (McCarthy et al., 2019). In clinical practice, several treatment strategies have been used to mitigate the AMI cardiac damage. However, further optimized therapies are crucial to minimize myocardial cell damage and maintain cardiac function. Numerous studies have demonstrated that exosomes play important roles in most biological processes, recognizing exosomes as novel candidates in the treatment of heart disease (Tian et al., 2019). Commonly, exosomes can be transported between cells, carrying messenger RNAs (mRNAs), microRNAs, and proteins. A previous study reported that AMI causes changes in circulating miRNAs expression. Intriguingly, AMI-derived exosomes can carry and liberate miRNAs to nearby cells. Additionally, the miRNA levels in exosomes are markedly altered (Sahoo and Losordo, 2014). Cheng et al. observed that miR-1 and miR-208 were significantly increased in exosome-injected animals with AMI (Cheng et al., 2012). Kuwabara et al. demonstrated that as a marker of cardiomyocyte death, circulating miR-133a localizes inside exosomes and is released post Ca^2+^ stimulation (Kuwabara et al., 2011). Many of these exosomal miRNAs can beneficially influence cardiac repair by protecting cardiomyocytes from AMI damage. Since miRNAs in exosomes play a crucial role in myocardial cellular communication, it would be a target for drug therapy. However, few studies have been performed.

Along with being palatable, green tea has been known to help cure several ailments including arthritis, diseases of the nervous system, cancer, and cardiopathy (Yang et al., 2018). Epigallocatechin gallate (EGCG), the main constituent in green tea, has been speculated as a potential therapeutic drug in hypoxia-reperfusion damaged cardiomyocytes by decreasing mitochondrial injury and peroxidation (Nan et al., 2019). Jang et al. revealed that EGCG upregulates miR-16 in the exosomes and the treatment of exosomes derived from EGCG-treated cells can inhibit tumor-associated macrophage infiltration and M2 polarization (Jang et al., 2013). We assumed that EGCG might regulate miRNAs, which can be transferred to extracellular *via* exosomes and affect the myocardial microenvironment to offer protection. If so, it will provide a promising candidate in the treatment of AMI.

Apoptosis and autophagy are two types of gene-regulated cell death involved in heart disease (Ye et al., 2018). Yang Y et al. showed that exosomal miR-30a was highly enriched in the serum of AMI patients, with increasing exosome release contributing to the restriction of autophagy (Yang et al., 2016). Coincidentally, our previous study confirmed that EGCG alleviated I/R injury in myocardial cells by regulating apoptosis and autophagy (Xuan and Jian, 2016). Furthermore, the beneficial effect of EGCG on attenuating mitochondrial impairment and myocardial apoptosis was associated with miR-30a levels (Zhang C. et al., 2019). Accordingly, we hypothesized that exosomal miR-30a could be a target of EGCG. The present study aimed to investigate whether exosomes derived from EGCG-treated cardiomyocytes attenuated AMI injury by regulating miR30a.

## Methods

The detailed experimental approach is presented in the **Supplementary Material**.

### Cell Culture and the Establishment of the AMI Model *In Vitro*

For all experiments, H9c2 cardiomyocytes were cultured in an exosome-depleted medium. The AMI model was established according to a previously described method (Zhang X. et al., 2019). Briefly, an exosome-depleted medium was replaced with glucose-free Dulbecco Eagle minimum essential medium (DMEM) to imitate ischemia. Then, the H9c2 cells were incubated in a hypoxic environment for 24 h, where normal air was replaced by a mixed gas of 5% CO_2_, 1% O_2_, and 94% N_2_. The normal cells were cultured under normoxic conditions with high sugar DMEM. The EGCG (25 µM), Z-VAD-FMK (an apoptosis inhibitor, 20 µM), and 3-MA (an autophagy inhibitor, 10 mM) groups were pretreated and incubated with cells for 4 h.

### Experimental Animals and AMI Model *In Vivo*

All animals were treated in accordance with the Guide for the Care and Use of Laboratory Animals, and all animal investigation protocols were approved by the institutional animal care and use committee of Guilin Medical University. All surgical procedures were performed under anesthesia, with every effort made to minimize suffering. Male Sprague-Dawley rats (150–200 g; Guilin Medical Laboratory Animal Center) were fasted overnight. Next, the corresponding EGCG (10 mg/kg), 3-MA (3.5 mg/kg), and Z-VAD-FMK (1 mg/kg) were injected into the sublingual veins 2 h before ischemia. The rats were anesthetized with sodium pentobarbital (40 mg/kg, intraperitoneally) and connected to a rodent ventilator, then left anterior-descending artery was ligated with a 7-0 silk suture (Finger et al., 2019), except in the sham group. The rats exhibited no signs of peritonitis, pain, or discomfort during the whole process. After 12 h of ischemia, a 1 mL blood sample was collected from the left ventricle and centrifuged at 3,600×g for 20 min to harvest sera. At the end of the experiment, the animals were euthanized using inhalational isoflurane (1.5–2%) and cervical dislocation was performed after deep anesthesia. When the animal's breathing stopped completely, the ischemic areas of the heart were immediately excised and stored at −80°C for analyses as described below.

### Co-Culture of Exosomes and Cells

To discover the role of exosomal miR30a in H9c2 cells, 50 µg/mL of exosomes (Exo) isolated from H9c2 and miR30a^KD^ cells and cultured either in normoxia (Exo^Nor^), hypoxia (Exo^Hypo^), or hypoxia + EGCG conditions (Exo^Hypo+EGCG^) were incubated with H9c2, miR30a^KD^, or miR30a negative control (miR30a^NC^) cells at 37°C for 24 h *in vitro*. Similarly, 50 µg/mL of exosomes (Exo) isolated from animal serum either from sham (Exo^Sham^), AMI (Exo^AMI^), or AMI + EGCG conditions (Exo^AMI+EGCG^) were also incubated with H9c2, miR30a^KD^, or miR30a negative control (miR30a^NC^) cells. Next, a cell hypoxia experimental model was established, while apoptosis and autophagy were measured separately.

### Statistical Analysis

All data are shown as the mean ± SD. Statistical analysis was performed using a one-way ANOVA followed by Bonferroni's multiple comparison tests. All analyses were performed using statistical software SPSS 21.0 (IBM Armonk, New York, USA). Statistical significance was defined as p < 0.05.

## Results

### EGCG Promoted Recovery of Cardiac Function and Attenuated Cell Damage

**Figure 1:**
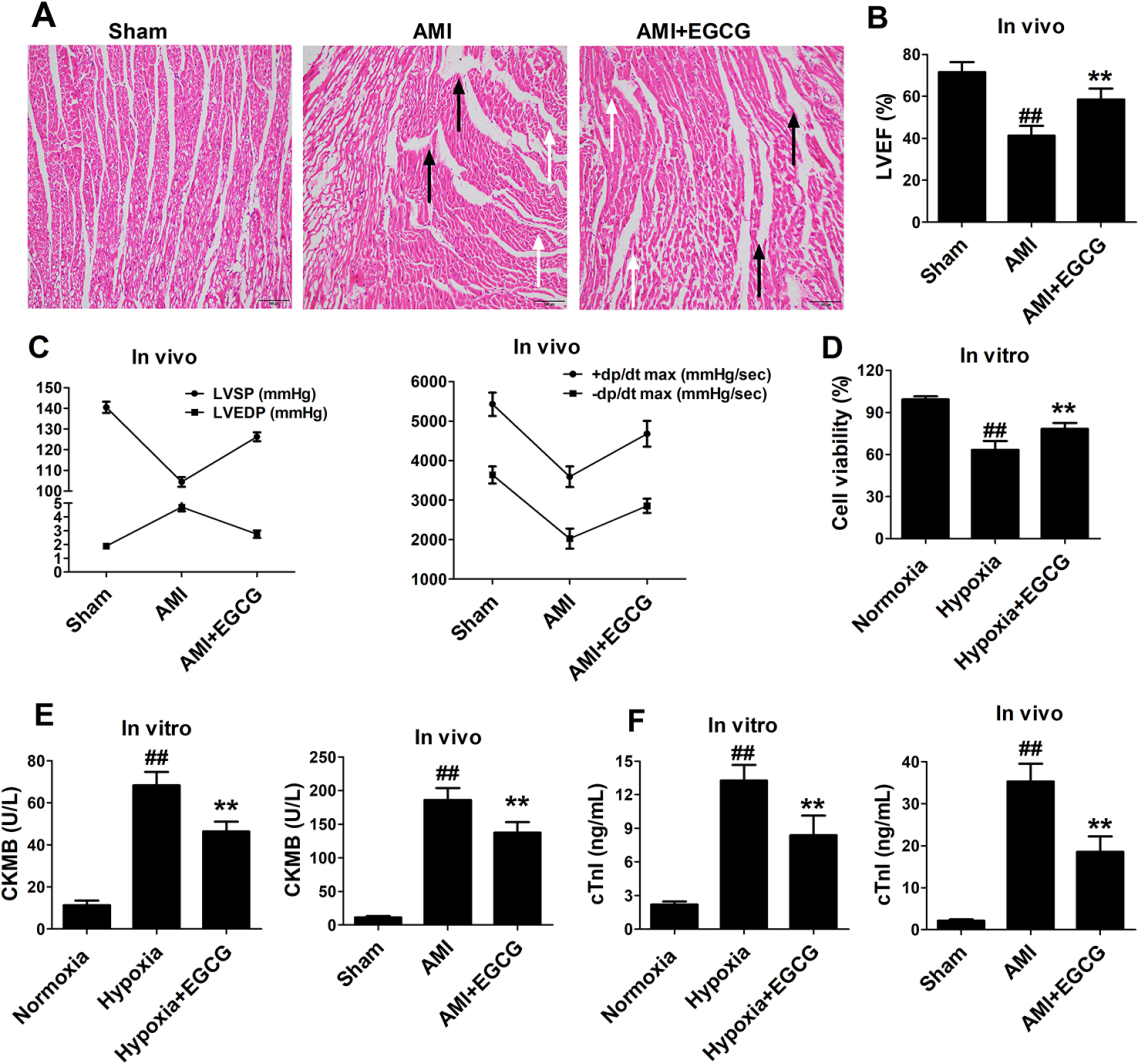
EGCG promoted recovery of cardiac dysfunction and attenuated cell damage. **(A)** Representative images of H&E-stained sections. Black arrow stands for infarct area, white arrow stands for border zone areas. **(B)** Left ventricular ejection fraction (LVEF). **(C)** Left ventricular systolic pressure (LVSP) and Left ventricular end-diastolic pressure (LVEDP), +dp/dt max and −dp/dt max. **(D)** Cell viability was determined by CCK-8 assay. **(E–F)** ELISA was performed to determine the expression levels of CK-MB, and cTnI myocardial injury markers in cell culture supernatant and serum. The data are presented as the means ± SD (n = 6 per group). ANOVA testing was performed; ^##^ *p* < 0.01 vs. sham (normoxia) group; **p < 0.01 vs. AMI (hypoxia) group.

Compared to the sham group, HE staining indicated that the AMI group demonstrated obvious histological damage in the infarct and border zone areas, presented as the appearance of necrotic tissue, greater disarray, and increased irregularity. However, the areas of necrosis were limited, and the myocardium was orderly arranged in the EGCG-treated group (**Figure 1A**). Furthermore, the left ventricular ejection fraction, left ventricular systolic pressure, and maximum rate of the ventricular pressure rise ( ± dp/dtmax) in the I/R group were significantly decreased, whereas left ventricular end-diastolic pressure was remarkably increased. Notably, EGCG pretreatment reversed these changes (*p* < 0.05) (**Figures 1B, C**). As expected, hypoxia caused decreased cell viability, while the cell viability was markedly elevated by EGCG pretreatment (*p* < 0.05) (**Figure 1D**). In addition, the release of cell-damaging factors, creatine kinase-MB (CK-MB) and troponin I (cTnI) was increased in the hypoxia (AMI) group. However, the increased levels were significantly abrogated in the hypoxia (AMI) + EGCG group (*p* < 0.05) (**Figures 1E, F**).

### EGCG Protected Cardiomyocytes Against AMI Injury by Regulating Apoptosis

**Figure 2:**
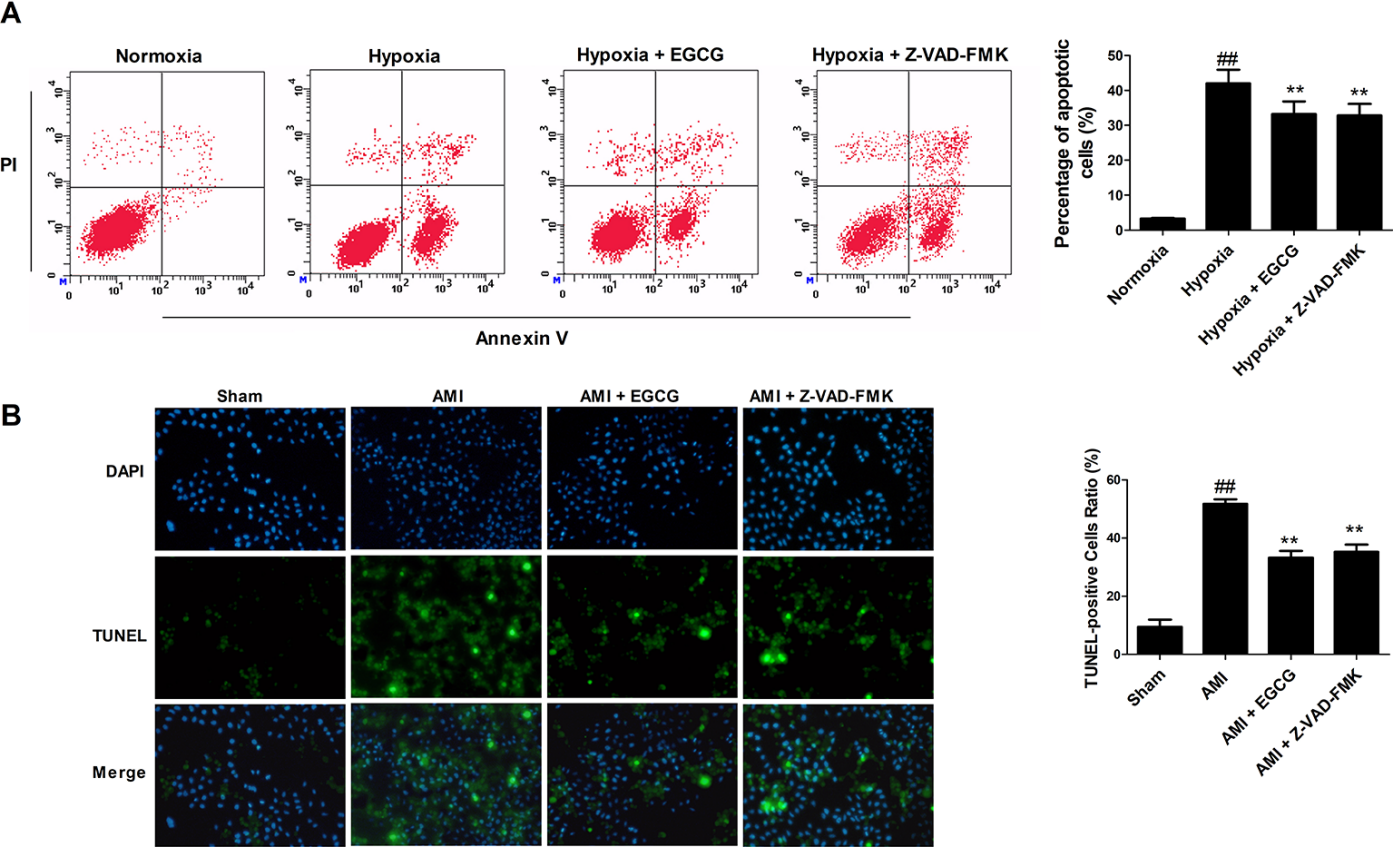
EGCG protected cardiomyocytes against AMI injury by regulating apoptosis. **(A)** The percentage of apoptotic H9c2 cardiomyocytes *in vitro* was detected by flow cytometry using Annexin V–fluorescein isothiocyanate (FITC) and propidium iodide (PI) staining. **(B)** Representative photomicrographs of terminal-deoxynucleoitidyl transferase mediated nick end labeling (TUNEL) staining *in vivo* (×400 magnification). Nuclei were stained with blue-fluorescent 4′,6-diamidino-2-phenylindole (DAPI). Values were expressed as mean ± SD (n = 6). ANOVA testing was performed; ^##^ *p* < 0.01 vs. sham (normoxia) group; **p < 0.01 vs. AMI (hypoxia) group.

Terminal-deoxynucleoitidyl transferase mediated nick end labeling staining *in vivo* and flow cytometry *in vitro* exhibited few apoptotic cells in the normoxic (sham) group. The percentage of apoptotic cells was markedly increased in the hypoxia (AMI) group. Preconditioning with EGCG or Z-VAD-FMK revealed a visual reduction of apoptosis-positive cells (*p* < 0.05 vs. model) (**Figure 2**). No significant difference was observed between the Z-VAD-FMK and EGCG pretreated groups (*p* > 0.05). The results suggested that EGCG protects AMI myocardial cells by inhibiting apoptosis.

### EGCG Maintained Cardiomyocytes Against AMI Injury by Regulating Autophagy

**Figure 3:**
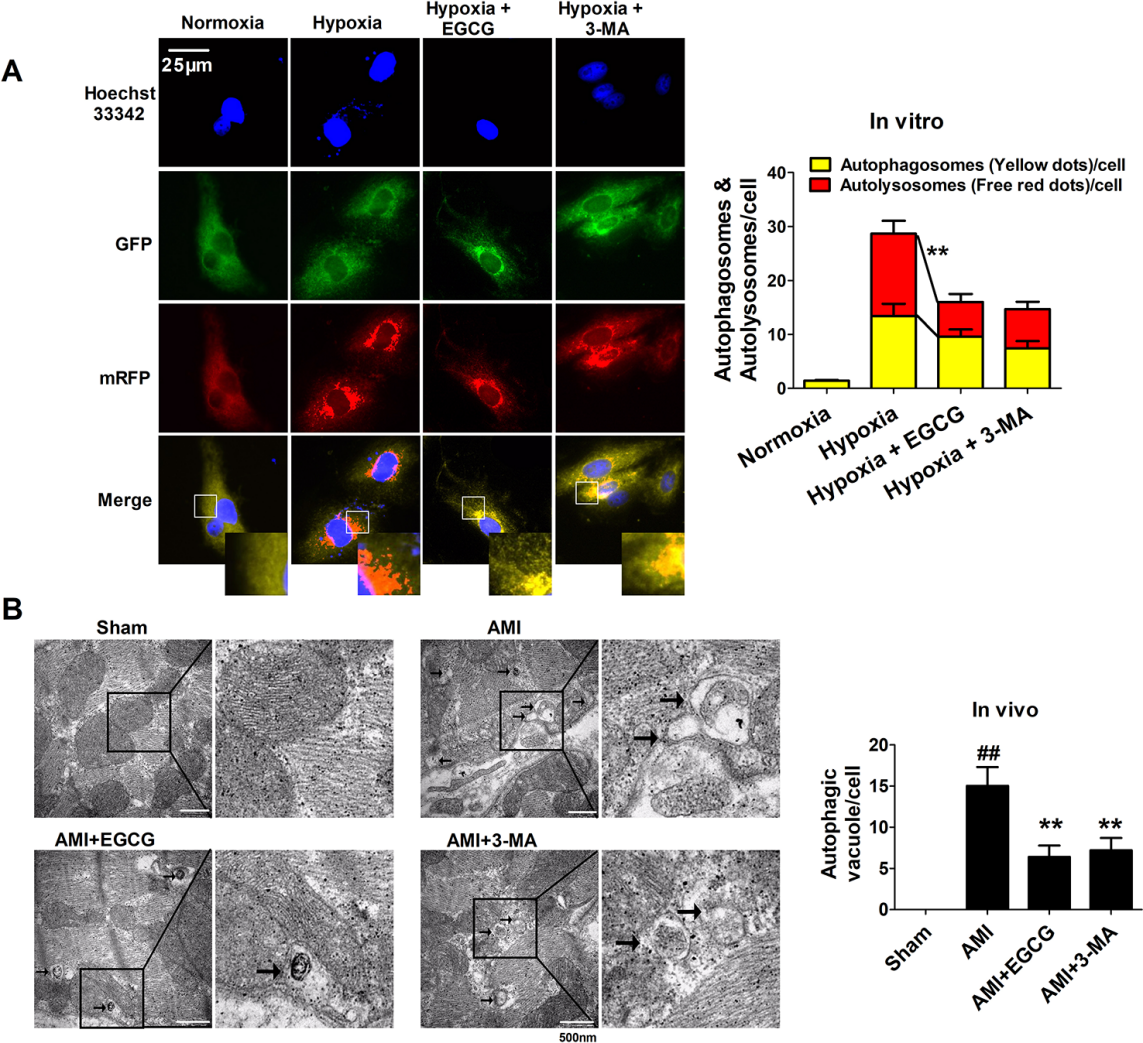
EGCG maintained cardiomyocytes against AMI injury by regulating autophagy. **(A)** Tandem mRFP-GFP-LC3B probe analysis of the autophagosome-lysosome fusion *in vitro*. Yellow dots represent the unfused autophagosome, whereas red dots denote the fusion, wherein autolysosomes are formed. At least 10 cells per group were counted randomly in three independent experiments. **p < 0.01 vs. other group. **(B)** Representative EM images were shown *in vivo*. The arrows depict autophagosome. Quantification of autophagic vacuoles was shown. Values were expressed as mean ± SD (n = 6). ANOVA testing was performed; ^##^ *p* < 0.01 vs. sham group; **p < 0.01 vs. AMI group.

To verify whether EGCG against AMI induced myocardial injury regulated autophagy, we designed and applied an mRFP-GFP-LC3B probe. The yellow fluorescence represents the production of autophagosomes, while the red fluorescence represents the autophagosome-lysosome. Hypoxia leads to a marked aggregation of red and yellow dots in the cells. EGCG pretreatment evidently decreased the red and yellow dots, as did pretreatment with the autophagy inhibitor 3-methyladenine (3-MA) (*p* < 0.01) (**Figure 3A**). Transmission electron microscopy (TEM) images demonstrated that typical autophagosomes (indicated by arrows) were present in the AMI cells. Meanwhile, a reduction in autophagosomes was observed in EGCG and 3-MA groups, suggesting the inhibition of autophagy (*p* < 0.01) (**Figure 3B**).

### Preparation and Characterization of Exosomes Secreted by Cardiomyocytes

**Figure 4:**
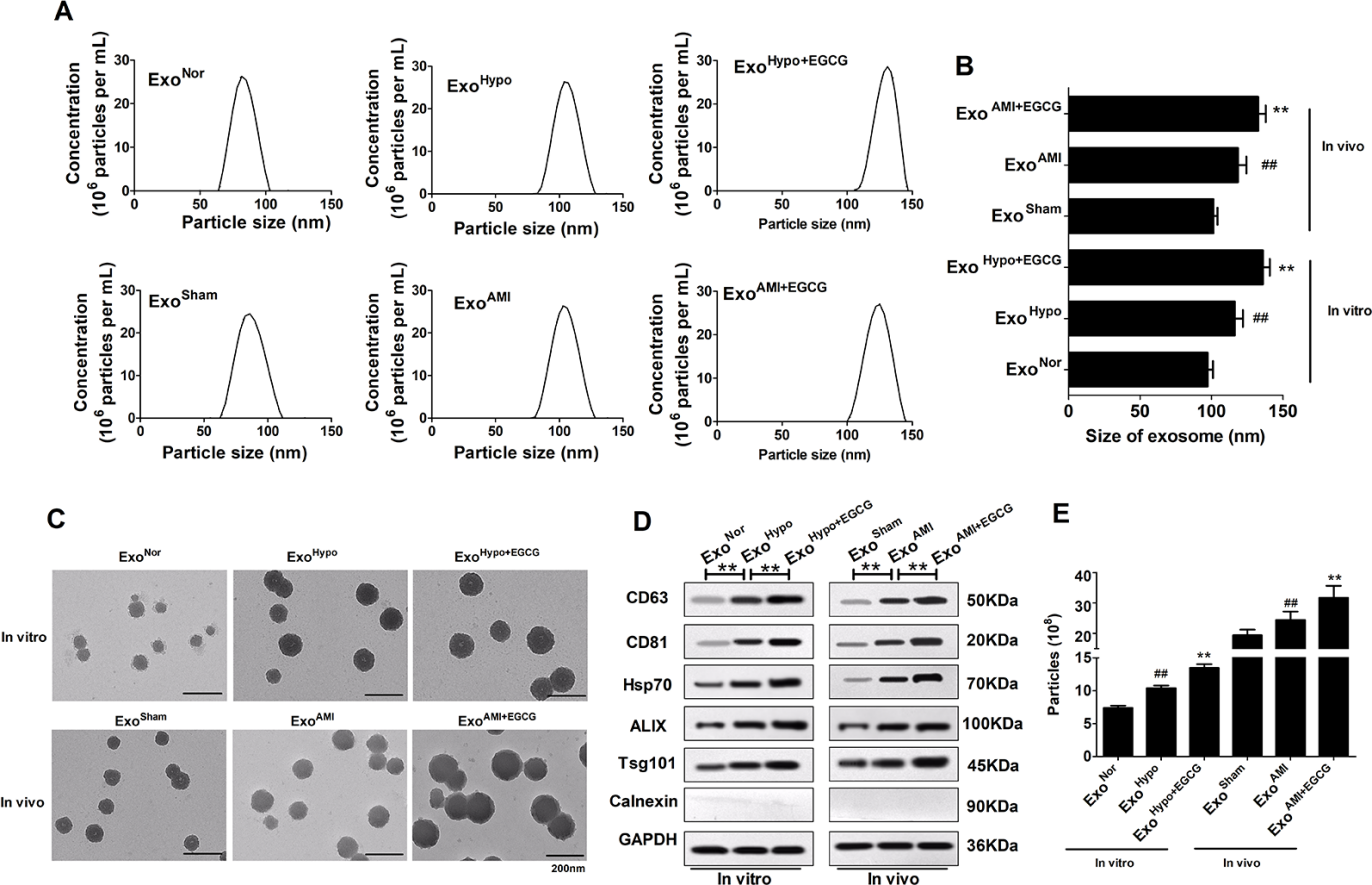
Preparation and characterization of exosomes secreted by cardiomyocytes *in vitro* and *in vivo*. **(A, B)** Representative graph of exosome concentration (E^6^ particles/mL) and size distribution of exosomes as measured by nanoparticle tracking analyses (NTA). ^##^ *p* < 0.01 vs. sham (normoxia) group; **p < 0.01 vs. AMI (hypoxia) group. **(C)** Representative images of exosomes released by cardiomyocytes visualized by TEM. Scale bars: 200 nm. **(D)** Representative images of western blotting to assess the presence of enriched proteins and calnexin in exosomes lysates. **(E)** The exosome concentrations *in vitro* and *in vivo* were recorded by NTA. Values were expressed as mean ± SD (n = 6). ANOVA testing was performed; **p < 0.01 vs. other group.

To determine whether EGCG modulated the release of exosomes, we extracted and isolated exosomes from their conditioned medium either in normoxia (Exo^Nor^), hypoxia (Exo^Hypo^), or hypoxia + EGCG conditions (Exo^Hypo+EGCG^) and animal serum either in sham (Exo^Sham^), AMI conditions (Exo^AMI^), or AMI + EGCG conditions (Exo^AMI+EGCG^). Nanoparticle tracking analyses and TEM were used to assess the morphology, size, and number of the vesicles. The exosome marker proteins (CD63, CD81, Hsp70, Alix, and TSG101) were typically enriched, while calnexin was absent, confirming that the exosomes were successfully extracted. On the contrary, the profile of exosomes in different groups was very similar, but the diameter differed, such as that of the Exo^Nor^ group was 97.37 ± 3.68 nm, that of the Exo^Hypo^ group was slightly larger at 116.28 ± 5.73 nm, and that of Exo^Hypo+EGCG^ was the largest at 135.93 ± 4.93 nm (*p* < 0.05) (**Figures 4A–C**). The levels of exosomal markers were enriched in Exo^Hypo^ but were less pronounced in Exo^Nor^, with enhanced expression demonstrated in Exo^Hypo+EGCG^ (*p* < 0.05) (**Figure 4D**). Additionally, the concentration of Exo^Hypo+EGCG^ was markedly higher than that of Exo^Hypo^ (*p* < 0.05) (**Figure 4E**). The character of exosomes secreted in the animal serum was similar to those in the cell culture medium.

### EGCG Upregulated miR30a Levels in AMI Cells and Exosomes

**Figure 5:**
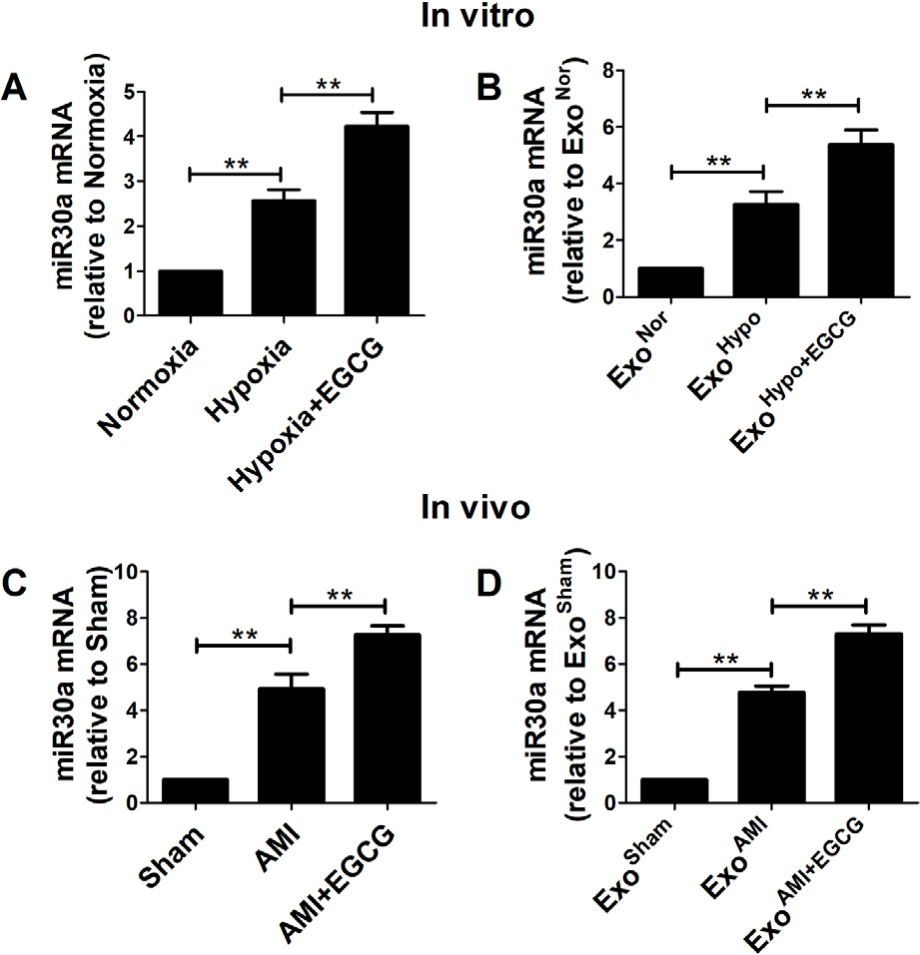
EGCG up-regulated miR30a levels in AMI cells and exosomes. Total RNA from cells and exosomes were extracted and subjected to qRT-PCR to evaluate the levels of miR30a *in vitro* **(A, B)** and *in vivo* **(C, D)**. Values were expressed as mean ± SD (n = 6). ANOVA testing was performed; **p < 0.01 vs. other group.

To further study the interaction between EGCG and exosomes/miR30a pathway in AMI injury, the total RNA was extracted and the miR30a expression in cells or the exosomal compartment was analyzed by qRT-PCR *in vitro* and *in vivo*. Compared to the hypoxia group, significant miR30a upregulation in both EGCG-treated cells and exosomes was observed *in vitro* and *in vivo* (*p* < 0.05) (**Figure 5**).

### Exosomes Derived From EGCG-Treated Cells Led to an Increase of miR30a Expression in a Paracrine Manner and Improved Cells Vitality

**Figure 6:**
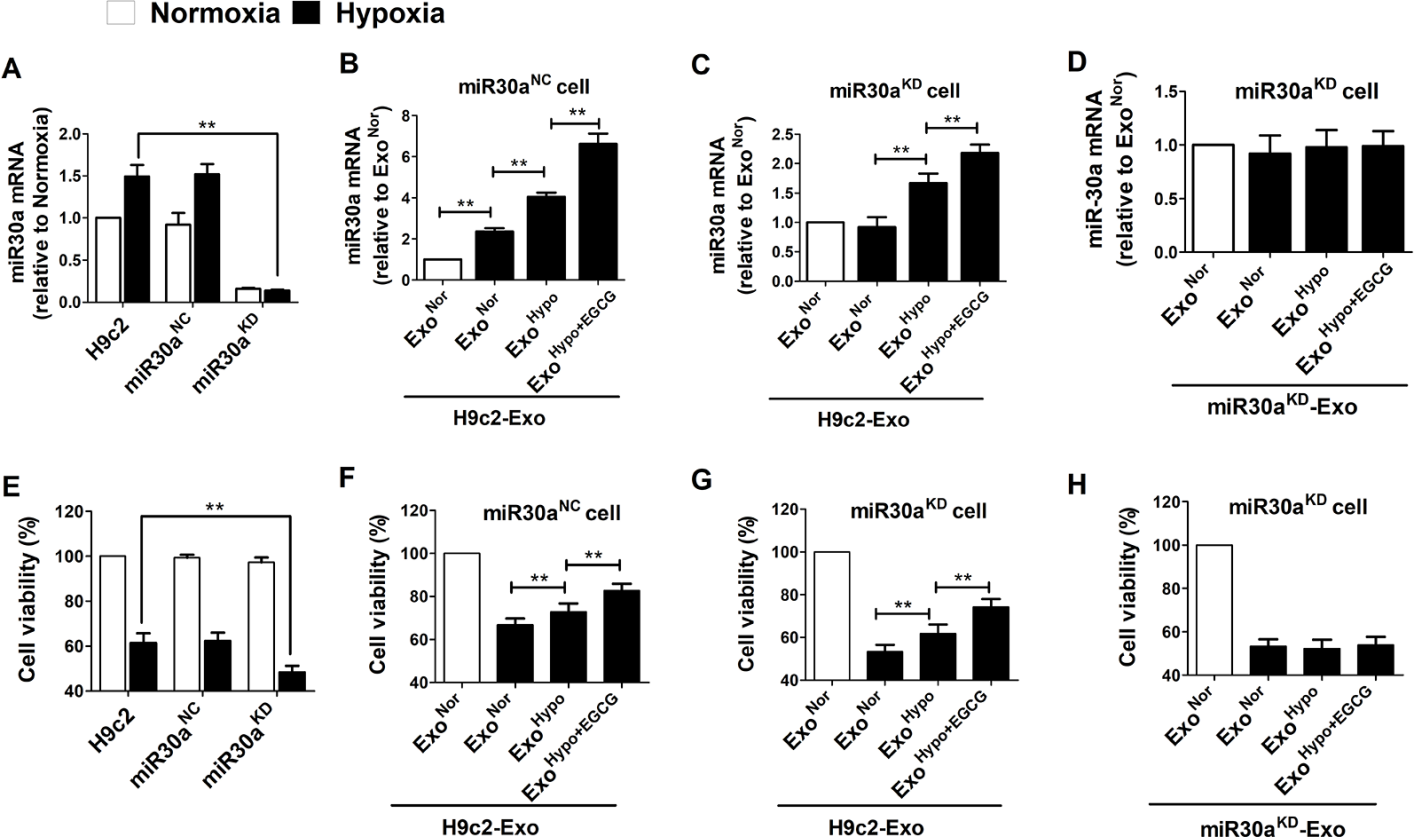
Exosomes derived from EGCG-treated cells led an increase of miR30a expression in a paracrine manner and improved cells vitality. **(A)** Total RNA from H9c2, miR30a^NC^ and miR30a^KD^ cells in normoxia and hypoxia conditions were extracted and subjected to qRT-PCR to detect the levels of miR30a. **(B)** MiR30a^NC^ cells were treated with exosomes (50 μg/mL) derived from H9c2 in normoxia conditions (Exo^Nor^), hypoxia conditions (Exo^Hypo^) or EGCG-treated hypoxia conditions (Exo^Hypo+EGCG^). The expression levels of miR30a in each group were determined with qRT-PCR. **(C)** MiR30a^KD^ cells were treated with exosomes (50 μg/mL) derived from H9c2 in normoxia conditions (Exo^Nor^), hypoxia conditions (Exo^Hypo^) or EGCG-treated hypoxia conditions (Exo^Hypo+EGCG^). The expression levels of miR30a in each group were determined with qRT-PCR. **(D)** miR30a^KD^ cells were treated with exosomes (50 μg/mL) derived from miR30a^KD^ cells in normoxia conditions (Exo^Nor^), hypoxia conditions (Exo^Hypo^) or EGCG-treated hypoxia conditions (Exo^Hypo+EGCG^). The expression levels of miR30a in each group were determined with qRT-PCR. **(E–H)** Cell viability in each group was determined by CCK-8 assay, the grouping was the same as A-D respectively. Values were expressed as mean ± SD (n = 6). ANOVA testing was performed; **p < 0.01 vs. other group.

To elucidate whether the protective effects of EGCG were related to exosomal miR30a regulation, the H9c2 cells were transfected with miR30a^KD^. In addition, the H9c2 cells were transfected with miR30a^NC^ as the blank control group. Compared to the H9c2 normoxic group, under hypoxia condition, the expression of miR30a was increased in H9c2 and miR30a^NC^ group but maintained a low expression in miR30a^KD^ group. However, cellular activity was decreased in all groups, especially in the miR30a^KD^ group (*p* < 0.05) (**Figures 6A, E**). This suggested that miR30a was a beneficial factor when ischemia occurred. Therefore, we tested whether exosomes derived from EGCG-treated H9c2 cells could suppress hypoxic damage, and if so, whether the mechanism of EGCG action is associated with the increased expression of exosomal miR30a.

Exo^Nor^, Exo^Hypo^, or Exo^Hypo+EGCG^ isolated from H9c2 *in vitro* and Exo^Sham^, Exo^AMI^ or Exo^AMI+EGCG^ isolated from animal serum *in vivo* were administered to miR30a^NC^ and miR30a^KD^ cells prior to the induction of hypoxia. The efficiency of H9c2 cells to uptake exosomes was more than 80% (**Figure S1**). Compared to Exo^Hypo^, treatment of miR30a^NC^ cells with exosomes from EGCG-treated H9c2 cells (Exo^Hypo+EGCG^) resulted in increased cellular activity and the concomitant accumulation of miR30a (*p* < 0.05) (**Figures 6B, F**). Moreover, when miR30a^KD^ cells were incubated with hypoxia-treated exosomes (Exo^Hypo^), miR30a level recovered and was more elevated with Exo^Hypo+EGCG^-treated, compared to the control group (cells were incubated with normoxia treated exosomes, Exo^Nor^) (**Figures 6C, G**). Exo^Hypo+EGCG^ pretreatment significantly increased miR30a^KD^ cell viability (*p* < 0.05). The exosomes obtained *in vivo* were co-incubated with miR30a^NC^ and miR30a^KD^ cells to further verify the reliability of the above experimental results (**Figure S2**).

To gain further mechanistic insights into the role of exosomal miR30a in the H9c2 cell hypoxia model, miR30a loss-of-function studies were performed. Exosomes isolated from miR30a^KD^ cells under different conditions were named miR30a^KD^-Exo^Nor^, miR30a^KD^-Exo^Hypo^, or miR30a^KD^-Exo^Hypo+EGCG^. miR30a expression did not increased in miR30a^KD^- exosomes and the cell viability was not elevated (*p* < 0.05) (**Figures 6D, H**).

### Upregulation of Exosomal miR30a by EGCG Pretreatment Markedly Alleviated Cardiac Damage Following AMI by Antagonizing Apoptosis and Excessive Autophagy

**Figure 7:**
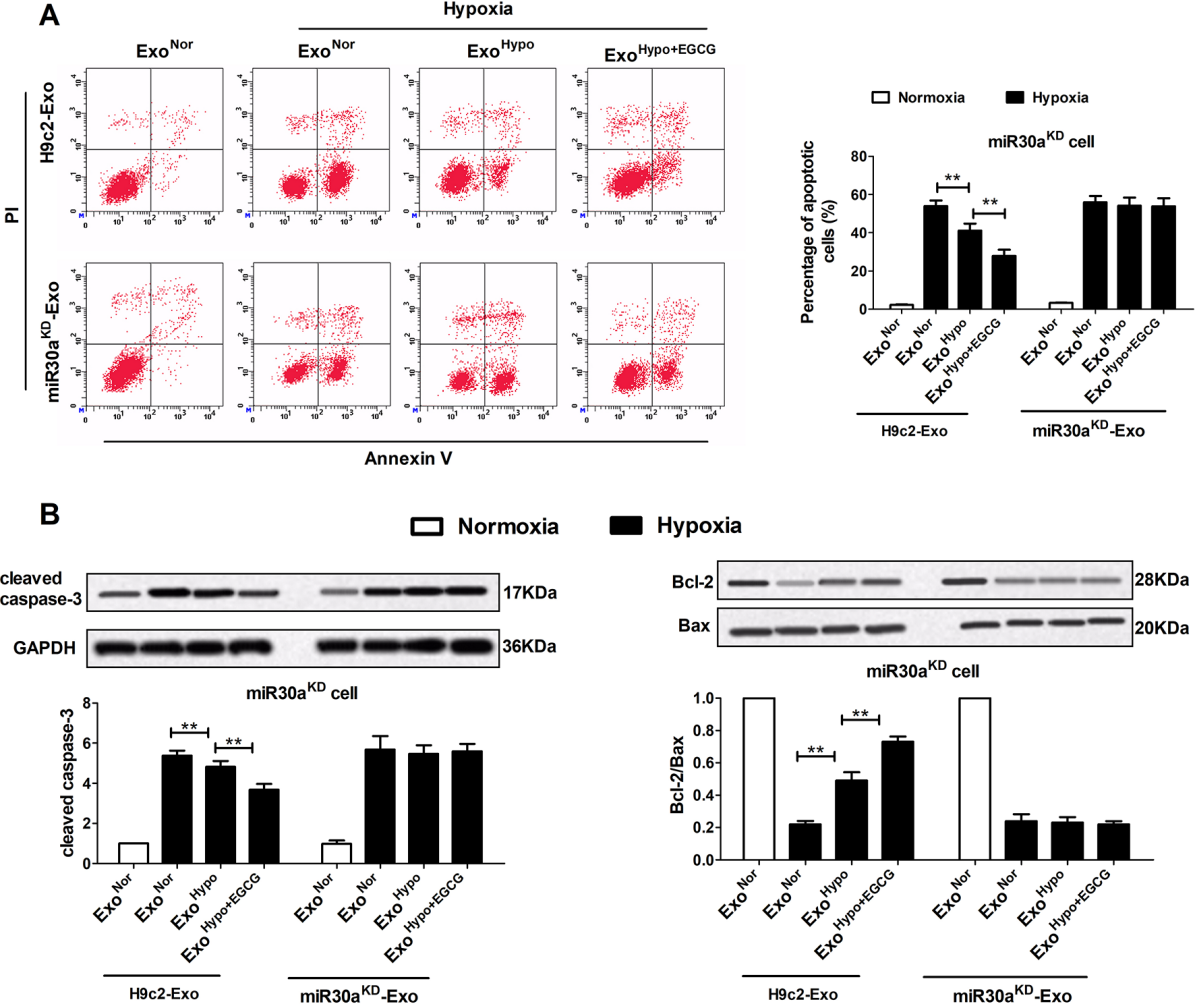
Up-regulation of exosomal miR30a by EGCG treatment markedly alleviated cardiac damage following AMI by antagonizing apoptosis. MiR30a^KD^ cells in normoxia or hypoxia conditions were treated with H9c2-Exo or miR30a^KD^-Exo (Exo^Nor^, Exo^Hypo,^ Exo^Hypo+EGCG^) (50 μg/mL). **(A)** The percentage of apoptotic cardiomyocytes was detected by flow cytometry using Annexin V-FITC and PI staining. **(B)** The levels of Bcl-2, Bax, and cleaved caspase-3 protein in cardiomyocytes were analyzed by western blotting. Values were expressed as mean ± SD (n = 6). ANOVA testing was performed; **p < 0.01 vs. other group.

**Figure 8:**
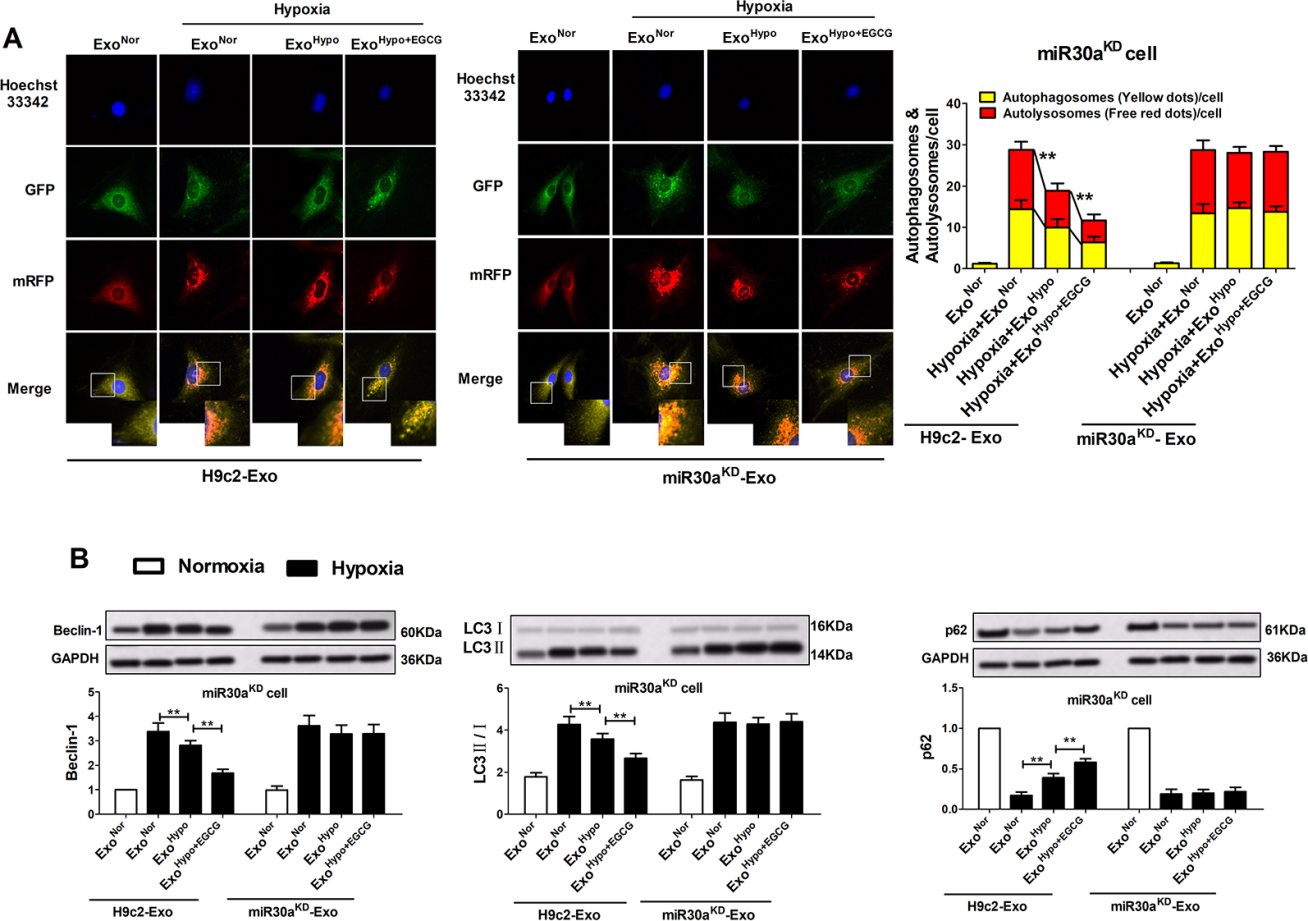
Up-regulation of exosomal miR30a by EGCG treatment markedly alleviated cardiac damage following AMI by antagonizing excessive autophagy. MiR30a^KD^ cells in normoxia or hypoxia conditions were treated with H9c2-Exo or miR30a^KD^-Exo (Exo^Nor^, Exo^Hypo^, Exo^Hypo+EGCG^) (50 μg/mL). **(A)** Tandem mRFP-GFP-LC3B probe analysis of the autophagosome-lysosome fusion. Yellow dots represent the unfused autophagosome, whereas red dots denote the fusion, wherein autolysosomes are formed. **(B)** The levels of Beclin-1, LC3 and p62 protein in cardiomyocytes were analyzed by western blotting. Values were expressed as mean ± SD (n = 6). ANOVA testing was performed; **p < 0.01 vs. other group.

To confirm the therapeutic potential of Exo^Hypo+EGCG^ (Exo^AMI+EGCG^) and examine whether exosomes from EGCG-treated myocardial cells could inhibit AMI injury by upregulating exosomal miR30a expression, we measured the apoptotic rate and the level of apoptosis-related proteins including caspase-3, Bax, and Bcl-2. Consistent with the cell counting kit-8 (CCK-8) data (**Figures 6F, H**; **Figures S2B, D**), incubation of miR30a^KD^ cells with Exo^Hypo+EGCG^ (Exo^AMI+EGCG^) resulted in the suppression of the apoptotic rate, caspase-3, and Bax, and an elevation of Bcl-2 (*p* < 0.05) (**Figures 7**, **S3**). However, the above effects of Exo^Hypo+EGCG^ were eliminated by incubation of miR30a^KD^ cells with miR30a^KD^-Exo^Hypo+EGCG^ (*p* > 0.05) (**Figure 7**). In addition, we examined the autolysosome formation and the level of autophagy-related proteins including Beclin-1, LC3, and p62. Similarly, incubation of miR30a^KD^ cells with Exo^Hypo+EGCG^ (Exo^AMI+EGCG^) isolated from *in vitro* and *in vivo* induced the suppression of autolysosome formation, Beclin-1, LC3 and elevation of p62 (**Figure 8**, **S4**); however, miR30a^KD^ -Exo^Hypo+EGCG^ did not demonstrate the same effect (**Figure 8**).

Together, these results suggested that EGCG pretreatment upregulated exosomal miR30a, as well as inhibited apoptosis and excessive autophagy, which contributed to the suppression of cell damages in AMI. This study could represent a new molecular pathway by which EGCG fulfills its anti-AMI potential by manipulating the myocardial cell microenvironment.

## Discussion

To improve the clinical treatment of AMI, an in-depth understanding of the pathophysiology and a search for new therapeutic targets are crucial. In the present study, we focused on manipulating the myocardial cell microenvironment to explore the cardioprotective mechanism of EGCG in AMI injury by reducing apoptosis and autophagy.

AMI causes serious cardiac dysfunction or heart failure (Walaszczyk et al., 2019). The early treatment of AMI is crucial for a favorable prognosis in patients. Notably, percutaneous coronary intervention has been adopted to recover blood perfusion at the ischemic site. However, subsequent reperfusion aggravates the myocardial cell injury. Thus, drug therapy would be an important part of the therapeutic strategies used to treat AMI more effectively and safely. Currently, myocardial infarction marker enzymes, CK-MB and cTnI, are predominant serum biomarkers used in the clinical diagnosis of AMI (Vylegzhanina et al., 2019). To confirm the successful establishment of the AMI in our model, we detected the cardiac function and the levels of CK-MB and cTnI. Our experimental results indicated that EGCG increased cell viability, supported myocardial fiber recovery, significantly ameliorated the cardiac function, and decreased CK-MB and cTnI release (**Figure 1**).

Apoptosis and autophagy are two pivotal events of programmed cell death in the pathological mechanisms of AMI injury (Bao et al., 2018). Cardiomyocyte apoptosis and autophagy occur during sustained and severe ischemia, usually involving myocardial pump failure (Guo et al., 2019). Studies have reported that autophagy plays a dual role, an opposite role in heart disease, whereas excessive autophagy resulting from prolonged hypoxia may induce cell death and cardiac dysfunction (Jian et al., 2015). Hence, reducing the magnitude of apoptosis and autophagy in cardiomyocytes could help prevent cell death. Our study revealed that pretreatment with EGCG significantly weakened apoptosis and excessive autophagy to attenuate cardiomyocyte injury *in vitro and in vivo* (**Figures 2** and **3**).

Currently, the potential cellular mechanisms leading to ischemic injury require further elucidation, along with the effects of EGCG on upstream targets of apoptosis and autophagy. Changes in the cellular microenvironment may be a critical determinant of AMI progression (Hu et al., 2018). Some studies uncovered the potential function of exosomes derived from various cells to regulate multiple processes, such as immune responses, tumor cell development, and cardiomyocyte survival, by mediating the communication among cells (Steinbichler et al., 2019). Exosomes, with a diameter of 50–150 nm, can be secreted from cardiomyocytes in an inducible manner and play a critical role in bridging the contact between cardiomyocytes, by carrying and releasing cytokines, trophic factors, and signaling molecules in cardiovascular disease (Cheng M. et al., 2019; Masaoutis et al., 2019). For example, Ribeiro-Rodrigues TM et al. established that exosomes secreted by cardiomyocytes under ischemic conditions promoted cardiac angiogenesis (Ribeiro-Rodrigues et al., 2017). Liu Z et al. confirmed that exosomes originating from adipose-derived stem cells can protect cardiomyocytes from oxidative stress (Liu et al., 2019). Hence, drugs regulating the release of exosomes may represent a promising therapeutic option for the treatment of myocardial infarction. Reportedly, ingested exosomes can transfer the intravesicular content, including miRNAs, into the host intracellular environment, subsequently modulating the cellular activities of the recipient cells (Meng et al., 2019). Our study characterized the exosomes derived from EGCG pretreatment cardiomyocytes in the AMI model *in vitro* and *in vivo*. In cell cytoplasm (animal serum), compared to the AMI group, the diameter and concentration of EGCG-derived exosomes were significantly increased, along with the expression of specific marker proteins (**Figure 4**). We postulated that the huge difference in size and concentration of EGCG-derived exosomes could be attributed to the increased cargo, mainly exosomal miRNAs and mRNAs. In other words, the experimental results indicated that EGCG affected the release of exosomes from ischemic myocardial cells and exploring the benefits or drawbacks of this effect was valuable.

In recent years, exosomal miRNAs have received substantial attention. Studies demonstrated that miR-30a is an important and promising biomarker in left ventricular dysfunction after AMI (Maciejak et al., 2018). Yang et al. discovered for the first time that miR30a is highly expressed in the myocardium and efficiently transferred *via* exosomes between hypoxic cardiomyocytes (Yang et al., 2016). Coincidently, our preliminary results demonstrated that miR30a is a key regulator of EGCG action in ischemic reperfusion injury (Zhang C. et al., 2019). Based on previous studies, we proposed that EGCG achieved an improved effect on cardiac recovery following AMI by specifically targeting the regulatory exosomal miR30a. In the current study, we analyzed the miR30a content of cells and exosomes *in vitro* and in the *in vivo* model of AMI with or without EGCG pretreatment. Compared to the AMI group, the miR30a levels in cells and exosomes were markedly elevated with EGCG pretreatment (**Figure 5**). However, this was insufficient to confirm that miR30a in Exo^Hypo+EGCG^ or Exo^AMI+EGCG^ reinforced ischemic heart repair and decreased cardiomyocyte injury in AMI. To further verify the interaction between miR30a and exosomes in hypoxia-induced cardiomyocytes, miR30a^KD^ was transfected into H9c2 cells and exosomes extracted *in vivo* and *in vitro* were incubated with H9c2, miR30a^NC^, or miR30a^KD^ cells.

As expected, compared to the H9c2 hypoxia group, miR30a^KD^ decreased cell viability. Accordingly, we observed that, compared with the Exo^Hypo^ (Exo^AMI^) hypoxic group, preconditioning with Exo^Hypo+EGCG^ (Exo^AMI+EGCG^) in miR30a^NC^ cells improved the hypoxic cell survival rate and miR30a expression. Notably, in the miR30a^KD^ cells, the results were in concordance with miR30a^NC^ cells. This could indicate that exosomal miR30a exerts a cardioprotective function in myocardial infarction by reacting in host cells (**Figures 6**, **S2**). In contrast, when miR30a^KD^-Exo^Hypo+EGCG^ was incubated with miR30a^KD^ cells, the expression of miR30a and cell survival rate did not increase (**Figure 6**). This result indicated that when the expression of exosomal miR30a was inhibited, the protective effect of EGCG in hypoxic injury was attenuated. That is, the beneficial function of EGCG was associated with the regulation of exosomal miR30a expression.

As previously mentioned, EGCG demonstrated the potential to treat AMI through anti-apoptosis and anti-autophagy. Coincidentally, exosomal miR30a inhibits autophagy and plays a protective role in heart disease (Yang et al., 2016). Hence, we assumed that the protective effect of EGCG was associated with the modulation of exosomal miR30a expression by anti-apoptosis and anti-autophagy and this effect had not yet been reported. Disrupting the balance between anti-apoptotic Bcl-2 and pro-apoptotic Bax leads to apoptosis, and therefore, the Bax/Bcl-2 ratio is an important indicator of apoptosis. The activation of the caspase-3 pathway is the main cause of apoptosis (Lu et al., 2019). microRNAs are small endogenous noncoding RNAs that act as gene regulators at the transcriptional and posttranscriptional levels by binding to 3′-untranslated regions (UTRs) (Shah et al., 2019). Studies have indicated that miR-30a-5p enhanced paclitaxel sensitivity in non-small cell lung cancer by inducing apoptosis through Bcl-2 inhibition, and miR-30a expression negatively correlated with Bcl-2 (Xu et al., 2017). Thus, we concluded that Bcl-2 could be a direct target of miR-30a to inhibit apoptosis. Moreover, LC3 and Beclin-1 are crucial cytoplasmic proteins required for the formation of the autophagosome (Tan et al., 2018) and p62 can be detected as an autophagosome and lysosome fusion marker protein (Peng et al., 2019). They are positively or inversely related to autophagy activity. Several studies have shown that cell autophagy may be attenuated by miR-30a-mediated translational control of Beclin-1, and miR-30a directly binds to the 3′-UTR of Beclin-1 gene (Bi et al., 2019). Therefore, Beclin-1 is also a direct target of miR-30a to inhibit autophagy.

In our experiment, western blotting was used to evaluate the expression apoptosis and autophagy-related proteins, including Bcl-2 and Beclin-1. The apoptosis rate and autophagosome-lysosome fusion were evaluated by flow cytometry and laser scanning confocal microscopy, respectively. Our research demonstrated that in the miR30a^KD^ cells, compared to the Exo^Hypo^ (Exo^AMI^) hypoxia group, treatment with Exo^Hypo+EGCG^ (Exo^AMI+EGCG^) suppressed the hypoxic cell apoptosis rate, autophagosome-lysosome fusion, and repressed the expression of cleaved caspase-3, Bax/Bcl-2 ratio, Beclin-1, LC3-II and increased the expression of p62 (**Figures 7** and **8**, **S3** and **S4**). Conversely, on treatment with miR30a^KD^-Exo^Hypo+EGCG^, the above regulatory effect disappeared. These results indicated that EGCG could modulate the miR30a localization within exosomes and suppress AMI-induced apoptosis and autophagy of cardiomyocytes (**Figures 7** and **8**). Collectively, these preliminary results confirmed that enhancing exosomal miR30a and inhibiting the miR30a downstream target gene, Beclin-1 or Bcl-2, might mediate the anti-AMI mechanism of EGCG, necessitating further research.

## Conclusions

**Figure 9:**
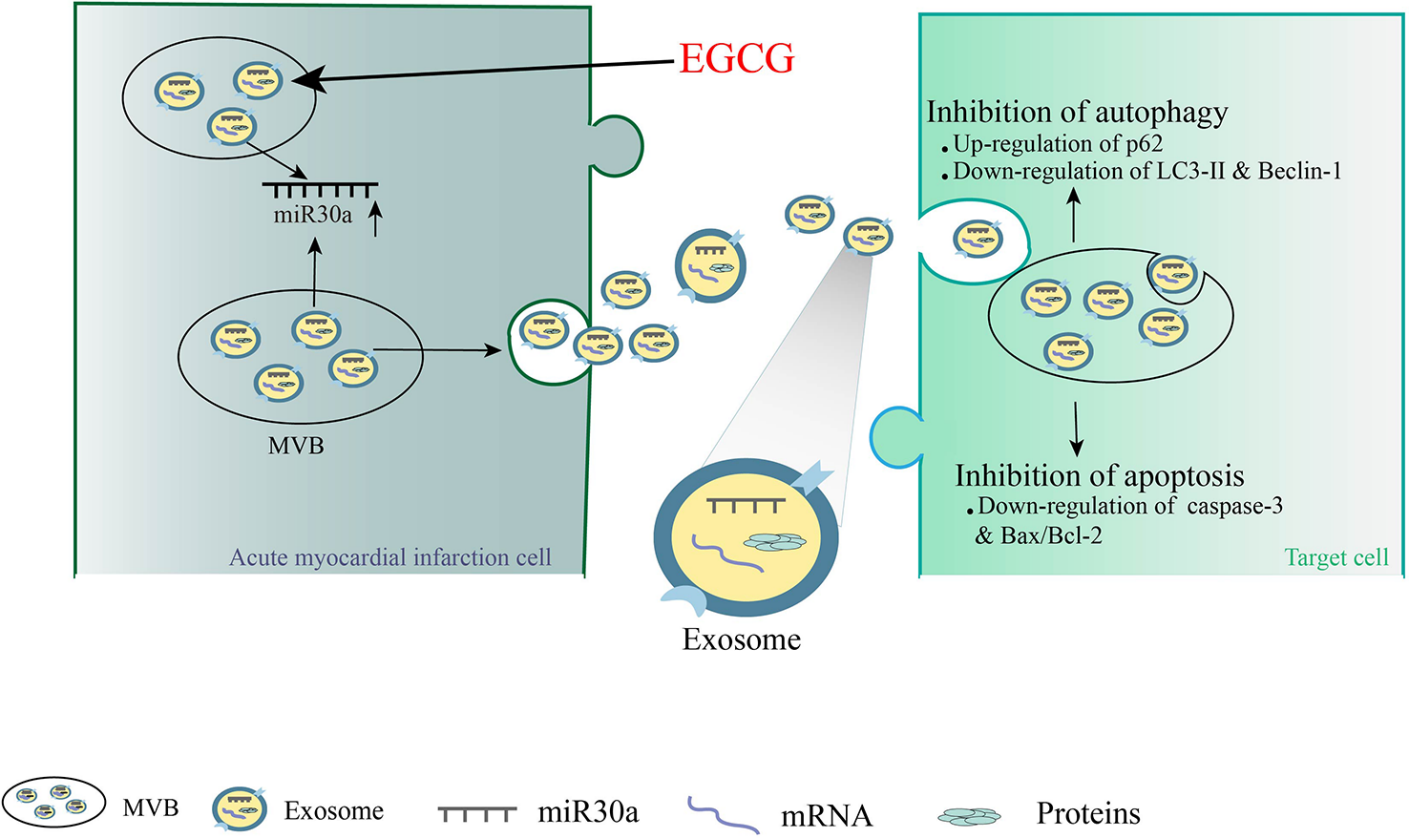
A novel mechanism demonstrating that EGCG can protect the ischemic heart by promoting the release of exosomal carriers from ischemic cardiomyocytes *via* anti-apoptosis and anti-autophagy.

In summary, we revealed that EGCG influences AMI injury in cardiomyocyte microenvironment by upregulating miR30a, which was transferred to adjacent cardiomyocytes *via* cell-derived exosomes. Our studies highlighted a novel mechanism demonstrating that EGCG can protect the ischemic heart by promoting the release of exosomal carriers from ischemic and hypoxic cardiomyocytes *via* anti-apoptosis and anti-autophagy. Furthermore, it could offer a novel concept for the development of EGCG as a potential drug in anti-AMI therapy (**Figure 9**).

## Data Availability Statement

All datasets generated for this study are included in the article/**Supplementary Material**.

## Ethics Statement

The animal study was reviewed and approved by the institutional animal care and use committee of Guilin Medical University.

## Author Contributions

Study conception and design: JJ and CZ. Conducted experiments: CZ, XG, and RL. Analysis and interpretation of data: CZ. Drafting of manuscript and critical revision: CZ and JJ.

## Funding

This work was supported by Natural Science Foundation of China (No. 81560665, No. 81760726), Project of Natural Science Foundation of Guangxi, China (No. 2017 GXNSFAA 198244, 2018GXNSFAA050035). Special funding for 2017 Guangxi BaGui Scholars (2017143).

## Conflict of Interest

The authors declare that the research was conducted in the absence of any commercial or financial relationships that could be construed as a potential conflict of interest.

## Abbreviations

AMI, acute myocardial infarction; BSA, bovine serum albumin; CCK-8, cell counting kit-8; CKMB, creatine kinase-MB; cTnI, cardiac troponin-I; DAPI, 4′,6-diamidino-2-phenylindole; DMEM, Dulbecco Eagle minimum essential medium; ± dp/dt max, maximum rise/down velocity of left intraventricular pressure; EGCG, epigallocatechin gallate; Exo, exosomes; FITC, fluorescein isothiocyanate; Hypo, hypoxia; HRP, horseradish peroxidase; LC3, light chain 3; LVEDP, left ventricular end-diastolic pressure; LVEF, left ventricular ejection fraction; LVSP, left ventricular systolic pressure; 3-MA, 3-methyladenine; miR30a, microRNA-30a; miR30a^KD^, miR30a knock-down; mRNA, messenger RNA; NC, negative control; Nor, normoxia; OD, Optical density; PI, propidium iodide; qRT-RCR, quantitative real-time PCR; TBST, Tris-buffered saline containing 0.1% Tween-20; TEM, transmission electron microscopy.
