# Supplementary figures and images for: Exosomes Derived From Epigallocatechin Gallate-Treated Cardiomyocytes Attenuated Acute Myocardial Infarction by Modulating MicroRNA-30a
Source: Front Pharmacol. 2020 Feb 26;11:126. doi: 10.3389/fphar.2020.00126 (PMC7054242; doi:10.3389/fphar.2020.00126)

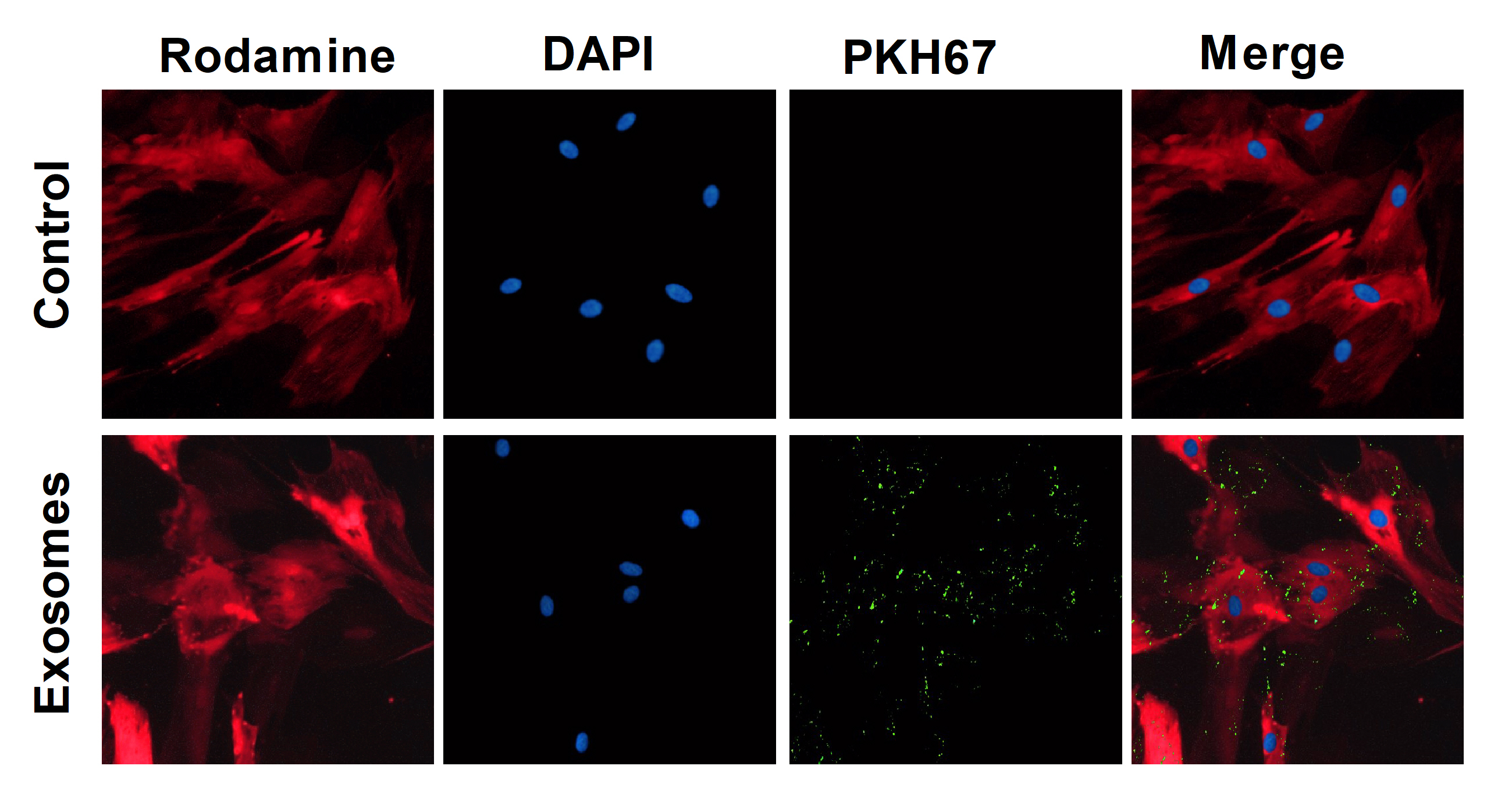

Supplement: Supplementary file 1 [file Image_1.jpeg]

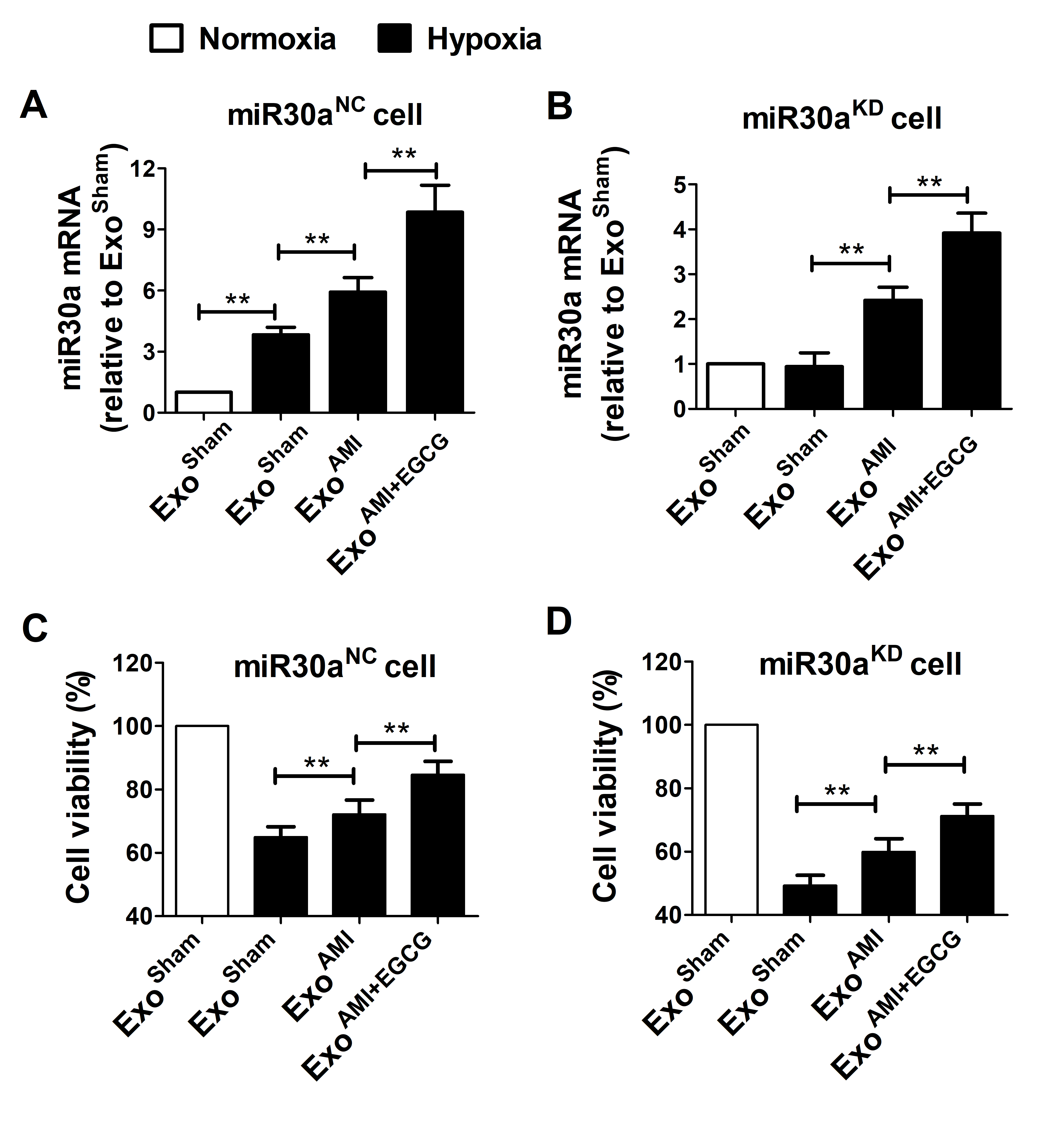

Supplement: Supplementary file 2 [file Image_2.jpeg]

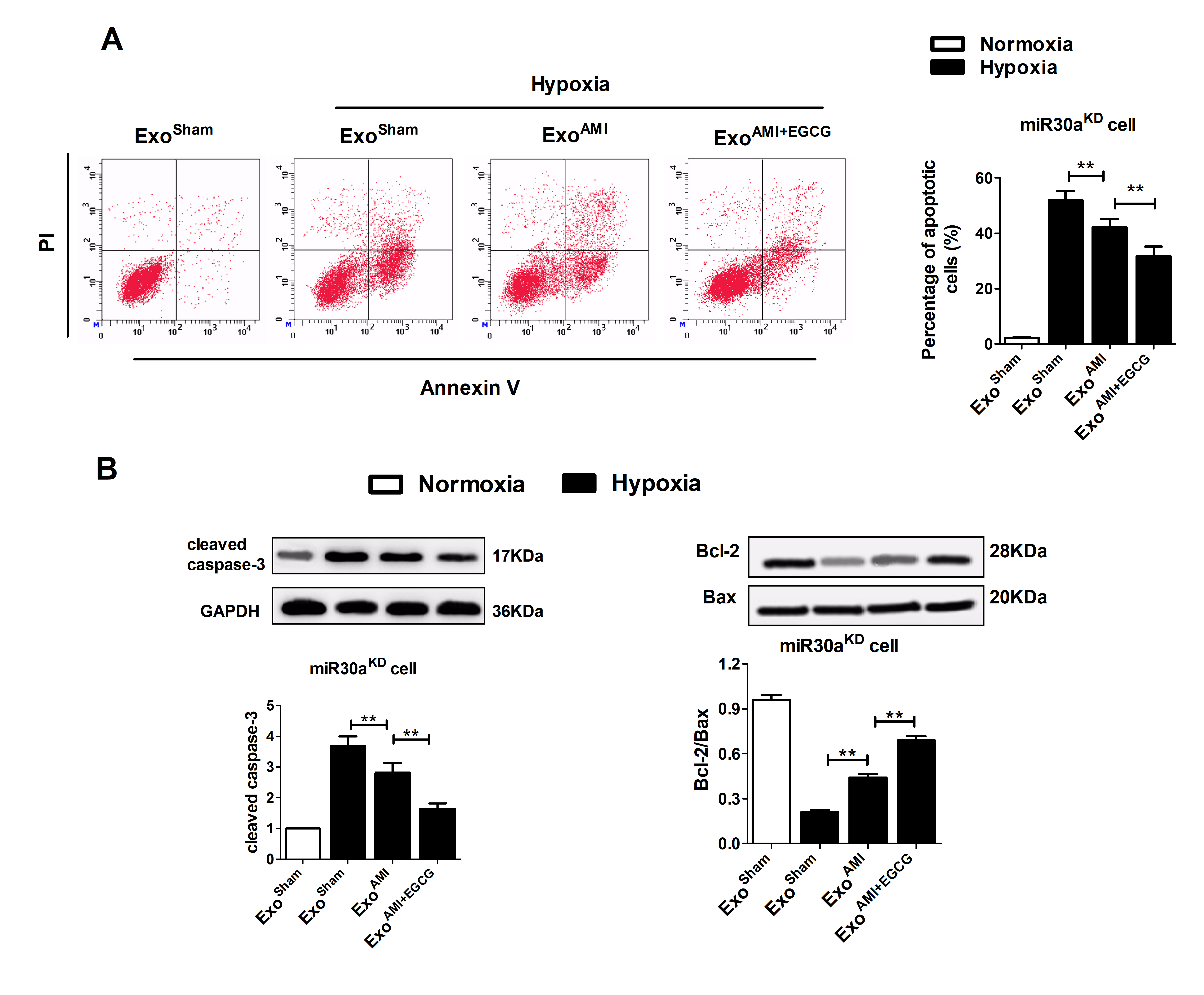

Supplement: Supplementary file 3 [file Image_3.jpeg]

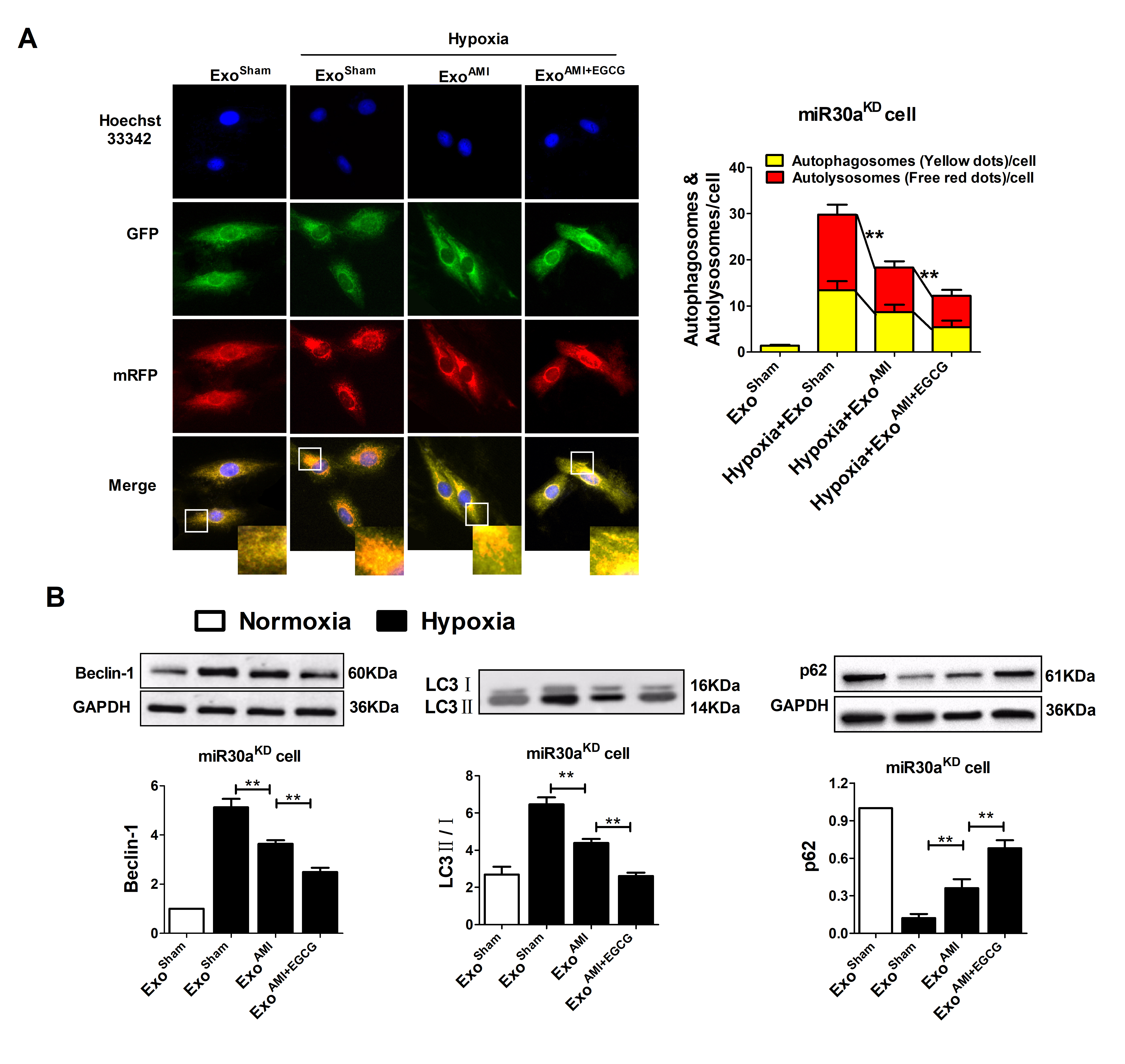

Supplement: Supplementary file 4 [file Image_4.jpeg]
